# Predictors of mortality among neonates hospitalized with neonatal sepsis: a case control study from southern Ethiopia

**DOI:** 10.1186/s12887-021-03049-5

**Published:** 2022-01-03

**Authors:** Tadele Bekele, Hailu Merga, Tamiru Tesfaye, Henok Asefa

**Affiliations:** 1 Durame General Hospital, Durame, SNNPR, Ethiopia; 2 Department of Epidemiology, Institute of Health, Jimma University, Jimma, Ethiopia; 3 Department of nursing, Ambo University, Woliso Campus, Ambo, Ethiopia

**Keywords:** Neonatal sepsis, Neonatal mortality, Epidemiology, Durame, Ethiopia

## Abstract

**Background:**

Neonatal sepsis, which resulted from bacterial, viral, and fungal invasions of the bloodstream, is the major cause of neonatal mortality and neurodevelopmental impairment among neonates. It is responsible for more than one-third of neonatal deaths in Ethiopia. Frequently neonates referred to health facilities are at high risk of death. Hence, assessing and preventing the predictors of mortality in neonatal sepsis helps to reduce the burden of neonatal mortality.

**Objectives:**

To determine predictors of mortality among neonates admitted with sepsis at Durame general hospital, southern Ethiopia, 2020.

**Methods:**

Institution-based unmatched case-control study was carried out from March 8 to 30, 2020, among 219 neonates in Durame general hospital in southern Ethiopia. Neonates admitted with sepsis and died were considered as cases and neonates admitted with sepsis and survived (discharged alive) as controls. Cases were selected by taking the deaths of neonates consecutively among those neonates admitted with the diagnosis of neonatal sepsis. The next immediate three corresponding controls were selected by lottery method from the Neonatal Intensive Care Unit (NICU) case registration book. Data was collected by using structured pretested checklists from neonates’ records and then entered into Epi data version 3.1 and exported to SPSS version 20. Logistic regression was used to identify the predictors of mortality. Statistical significance was declared at *P* < 0.05.

**Results:**

A total of 55 cases and 164 controls were included in this study. More than three quarters (81.8%) of cases had early onset sepsis. The multivariable logistic regression analysis showed that predictors of mortality in this study were; poor feeding [AOR = 4.15; 95% CI (1.64, 10.49)], respiratory distress [AOR = 2.72; 95% CI (1.31, 5.61)], estimated gestational age less than 37 weeks [AOR = 4.64; 95% CI (2.17, 9.91)], and convulsion [AOR = 3.13; 95% CI (1.12, 8.76)].

**Conclusion:**

This study showed that prematurity, convulsion, poor feeding, and respiratory distress were the predictors of sepsis-related neonatal mortality. It is important to pay attention to septicemic babies with any of the identified predictors to reduce sepsis-related mortality.

## Background

Neonatal sepsis is a clinical syndrome of bacteremia with systemic signs and symptoms of infection in the first 28 days of life. It can also result from viral and fungal invasions of the bloodstream [1]. It is considered as a case in the existence of suspected or confirmed infection in the neonate and includes many systemic infections of the newborn like septicemia, meningitis, pneumonia, arthritis, etc., but it does not encompass superficial mucocutaneous infections like thrush [2]. Neonatal sepsis can be early-onset sepsis (EOS) in the first week of life or late-onset sepsis which is usually infection occurring after 1 week [3].

The neonatal period subdivided into very early (birth to 24 h), early (birth to 7 days), and late (7 to 28 days) is the riskiest time for child survival [1]**.** During this period, marked physiologic transitions occur in all organ systems and they learn to respond to many forms of external stimuli, which implies that this period is a highly exposed time as they are completing many of the adjustments required for extra uterine survival [4]. In this period, the immaturity of the immune system, particularly in premature infants, confers distinctive clinical, physical, and outcome characteristics to infections compared with other age groups. Besides, inherent factors like poorly developed and immature skin barriers, mucosal defense mechanisms and blood-brain barriers contribute to the increased susceptibility of the neonates to infection. As a result, neonates are more vulnerable to a broad range of pathogens, including those of generally low virulence such as listeria, par echoviruses, or Candida [5].

Neonatal mortality, neonatal death within the first 28th day of life, is divided into early neonatal mortality, which is before the 7th day of life, and late neonatal mortality, which is occurring thereafter [6]. Sepsis is a major cause of neonatal mortality and neurodevelopmental impairment among neonates which results in death and major disability for 39% of those affected even with timely antibiotic treatment [7].

Globally, it is estimated that more than 1.4 million neonatal deaths annually are the consequence of invasive infections [8]. Infection-specific mortality varies by geographic region and neonatal risk factors like gestational age and body weight [9]. It contributes to nearly 30–50% of neonatal deaths in developing countries [10].

In Ethiopia, about 89,000 babies die every year in the first 4 weeks of life and it accounts for 44% of all deaths in children younger than 5 years of age [11]. The risk of death is highest in the first 24 h of life when more than half of deaths occur and about three-quarters of all neonatal deaths occur within the first week of life [6]. In 2019, Ethiopian mini demographic health survey (EDHS) reported neonatal mortality rate (NMR) as 30/1000 live births, which is almost similar to the 2016 report which was 29/1000 live births with no reduction [12]. One-third of these deaths is highly attributed to neonatal sepsis which is among the leading causes of neonatal death in Ethiopia [13]. A community-based study in the rural part of Ethiopia also reported sepsis as the leading cause of neonate death [14].

According to the 2016 EDHS result, there was a great regional variation in neonatal mortality and South Nation Nationalities and People Region were among the high mortality areas with 35 losses per 1000 live births [11]. There are studies gaps on determining risk factors for cause-specific neonatal deaths especially sepsis-related mortalities in neonatal intensive care units in our country context in general and in the study area in particular. Even though several studies have been conducted on neonatal sepsis, they mainly focus on epidemiology and didn’t address the outcome of the disease, and were mostly cross-sectional. Moreover, most of the time neonates referred to health facilities are at high risk of death and the sepsis-related factors behind this need to be investigated. Hence, this study focuses on determining predictors of sepsis-related neonatal deaths using methods of multiple logistic regression models that would help to guide health professionals and health policymakers to identify indicators for monitoring strategies and to apply appropriate preventive measures to decrease infant mortality.

## Methods

### Study setting and population

Institution-based unmatched case-control study was conducted among neonates admitted in.

NICU in Durame General Hospital (DGH) from March 8 to 30, 2020.

Durame General Hospital is one of the four hospitals in Kembata-Tambaro zone situated in South Nations Nationalities and Peoples’ regions, which is located 352 km from Addis Ababa, the capital. The hospital provides services for approximately 1.5 million people in its catchment area. The pediatrics department has 6 units that include outpatient and follow-up units, pediatrics emergency triage, assessment, and treatment unit (ETAT), neonatal intensive care unit (NICU), surgical unit, nutritional rehabilitation unit, and medical unit. The NICU is rendering service under critical newborn care unit, septic ward, kangaroo mother care (KMC), and mother side and receives 50 to 90 neonates monthly. Investigational modalities including electrocardiography (ECG), ultrasound, x-ray, and basic hematologic and chemistry tests were readily available.

In this study, cases were defined as neonates who were diagnosed with neonatal sepsis by the attending physician and died (registered as sepsis related death by attending physician). Also, in this study, hematological criteria along with the established IMNCI (Integrated Management of Neonatal and Childhood Illness), clinical features of neonatal sepsis were used to diagnose neonatal sepsis. Neonates in the presence of one or more of the established IMNCI clinical features [either of fever (> 38 °C) or hypothermia (< 36 °C), fast breathing (> 60 breath per minute), severe chest indrawing, poor feeding, the movement only when stimulated, convulsion, lethargic or unconscious] along with two of the hematological criteria; total leukocyte count(< 5000 or > 12,000 cells/m3, absolute Neutrophil count (< 1500 cells/mm3 or > 7500cells/mm3), erythrocyte sedimentation rate (ESR) (> 15/1 h) and platelet count (< 150 or > 440cells/mm3) were considered as having neonatal sepsis. Controls were defined as newborns admitted with a diagnosis of neonatal sepsis who were discharged alive (improved). The source population for both cases and controls were the whole neonates admitted to the Neonatal Intensive Care Unit (NICU) with the diagnosis of either early-onset or late-onset neonatal sepsis in the hospital. All neonates with features of sepsis (diagnosed as either LONS or EONS) who were admitted at the neonatal unit of Durame general hospital during the study period were included in the study. Neonates with incomplete records or chart didn’t available at the time of data collection and those neonates who were transferred to other hospitals or referred before the outcomes were assessed as well as neonates left the hospital against medical advises (withdrawn treatment) were excluded from the study.

### Sample size and sampling procedures

The sample size was determined by using Epi info version 7 stat calc programs by considering the following assumptions: Power 80, 95% confidence interval, 1:3 case to control ratio and using abdominal distension predictor, percent of controls who were exposed were 44.1%, odds ratio of 2.7 and 10% for incomplete records. This yields a sample size of 219 (55 cases and 164 controls).

Cases were selected by taking the deaths of neonates consecutively among those newborn infants admitted with the diagnosis of neonatal sepsis in the neonatal intensive care unit of the hospital until the sample size was achieved. This retrospective sampling covers a period of 1 year extended from January 1st to December 31st, 2019. The next immediate three corresponding controls were selected by lottery method from the NICU case registration book.

### Data collection tool and procedure

The lists of sampled neonatal septicemia patients’ medical record numbers were retrieved from the neonatal intensive care unit case registration book. The patients’ medical records (charts) were then collected from the hospital registry and checked for inclusion criteria. The medical records of eligible patients were reviewed and information was transferred into the data collection form (checklist) by the data collectors. The available data on the patient chart, NICU case registration book is observed, and an appropriate data extraction checklist is prepared in English. The checklist was adapted from the national neonatal registration book and previous related studies [13, 15]. Data were collected by two data collectors who have experience in data collection (one bachelor’s degree holder nurse and one specialty in neonatology nurse) and one supervisor (general practitioner) using the structured checklist. The data collectors were trained for 2 days on the objectives of the study, the selection of study participants (card), how to keep confidentiality of information, the contents of the questionnaire, and how to fill the data collection format by the principal investigator. Intensive supervision was maintained during the whole period of data collection. The pretest was performed on a 5% random sample of the registration form by the principal investigator to confirm the reliability of the data before the actual data collection. Proper coding and categorization of data were maintained for the quality of the data to be analyzed. Double data entry was used to ensure data quality.

### Data analysis

Data were checked for completeness and consistencies, and then it was coded and entered into EpiData version 3.1 and it was exported to SPSS windows version 20 for analysis. Descriptive statistical techniques were used to obtain summary values for cases and controls separately. Bivariate analysis was performed to identify the crude association between dependent and independent variables. Then variables that show association in the bivariate model (*p* < 0.25) were entered and analyzed in a multivariable logistic regression model by using a backward stepwise method to identify the predictors of sepsis-related neonatal mortality. Model fitness was evaluated through inspection of Hosmer–Lemeshow statistic test and provided (*P* = 0.514), which implies that the model’s estimates fit the data at an acceptable level. Odds ratio (OR) with a 95% confidence interval was used to assess the strength and direction of the association between factors associated with the occurrence of neonatal mortality. Statistical significance was declared at *P* < 0.05.

## Results

A total of 219 neonates (55 cases and 164 controls) who were admitted to NICU were included in this study. According to this study, the mean age of the study participants was 5.35 (S.D ± 4.77) days and the majority of cases 45 (81.8%) and controls 106 (64.6%)) were in the age group of less than 7 days (EONS). Most of the participants were from rural areas (61.8% of cases and 53.0% of controls) and more than half 28 (50.9% of cases and 91 (55.5%) of controls were males. Concerning the marital status of the mothers, 53 (96.4%) of cases and 160 (97.6%) controls were married. Regarding the bodyweight of the neonates’, the majority of the cases 36 (65.5%) had a bodyweight of less than 2.5 kg whereas the majority of the controls 102 (62.2%) were in the category of greater than 2.5 kg (Table 1).

**Table 1:** Socio demographic characteristics of the study participants in Durame general hospital, Kembata tambaro zone, southern Ethiopia, 2019 (*n* = 219)

Variable	Category	Case	Control	Total
Sex	Male	28(50.9)	91(55.5)	119(54.3)
	Female	27(49.1)	73(44.5)	100(45.7)
Residence of the mother	Urban	21(38.2)	77(47.0)	98(44.7)
	Rural	34(61.8)	87(53.0)	121(55.3)
Marital status of the mother	Single	2(3.6)	4(2.4)	6(2.7)
	Married	53(96.4)	160(97.6)	213(97.3)
Age	≤7 days	45(81.8)	106(64.6)	151(68.9)
	> 7 days	10(18.2)	58(35.4)	68(31.1)
Bodyweight	< 2.5 kg	36(65.5)	62(37.8)	98(44.7)
	≥2.5 kg	19(34.5)	102(62.2)	121(55.3)
Total		55	164	219

### Maternal related factors for mortality in neonatal sepsis

This study revealed that more than nine-tenths of mothers 52 (94.5%) of cases and 156 (95.1%) of controls ever had ANC service during their pregnancy of the current neonate. The proportion of mothers who got ANC service less than three times was higher in cases 10 (18.2%) than in controls 21 (12.8%). Similarly, the proportion of women who had multiple births (twin birth and above) was higher in cases 3 (5.5%) compared to controls 6 (3.7%). More than nine-tenths of women had given birth at a health facilities 51 (92.7%) of cases and 150 (91.5%) of controls. Also in this study 22 (40%) of the mothers among the cases and nearly three fourth among the controls 118 (72.0%) were in the gestational age group of 37–42 completed weeks (term) whereas the proportion of mothers with gestational age < 37 completed weeks was higher in cases 33 (60%) than controls 46 (28%). Regarding mode of delivery, about three fourth (73.2%) of controls and two-third (67.3%) of cases had a spontaneous vaginal delivery (Table 2).

**Table 2:** Maternal characteristics of neonates for the study of predictors of mortality in neonatal sepsis in durame general hospital, Kembata tambaro zone, southern Ethiopia, 2019

Variable	Category	Case	Control	Total
		Count (%)	Count (%)	
Parity of the mother	Primiparous	21(38.2)	85(51.8)	106(48.4)
	Multiparous	34(61.8)	79(48.2)	113(51.6)
Mode of delivery	SVD	37(67.3)	120(73.2)	157(71.7)
	C/S	13(23.6)	35(21.3)	48(21.9)
	Instrumental delivery	5(9.1)	9(5.5)	14(6.4)
Does the mother have ANC in recent birth	Yes	52(94.5)	156(95.1)	208(95.0)
	No	3(5.5)	8(4.9)	11(5.0)
No of ANC visit	1 to 3 visit	10(18.2)	21(12.8)	31(14.2)
	> 3 visit	42(76.4)	135(82.3)	177(80.8)
Type of pregnancy	Single	52 (94.5)	158 (96.3)	210 (95.9)
	Multiple	3(5.5)	6(3.7)	9(4.1)
Place of delivery	Health facility	51(92.7)	150 (91.5)	201(91.8)
	Home delivery	4 (7.3)	14 (8.5)	18(8.2)
Maternal HIV status	Positive	1 (1.8)	2 (1.2)	3(1.4
	Negative	51(92.7)	154 (93.9)	205 (93.6)
	Unknown	3(5.5)	8 (4.9)	11 (5)
Estimated gestational age in weeks	< 37 weeks	33(60.0)	46(28.0)	79(36.1)
	> = 37 weeks	22(40.0)	118(72.0)	140(63.9)
Total		55	164	219

### Neonatal related factors for mortality in neonatal sepsis

**Fig. 1:**
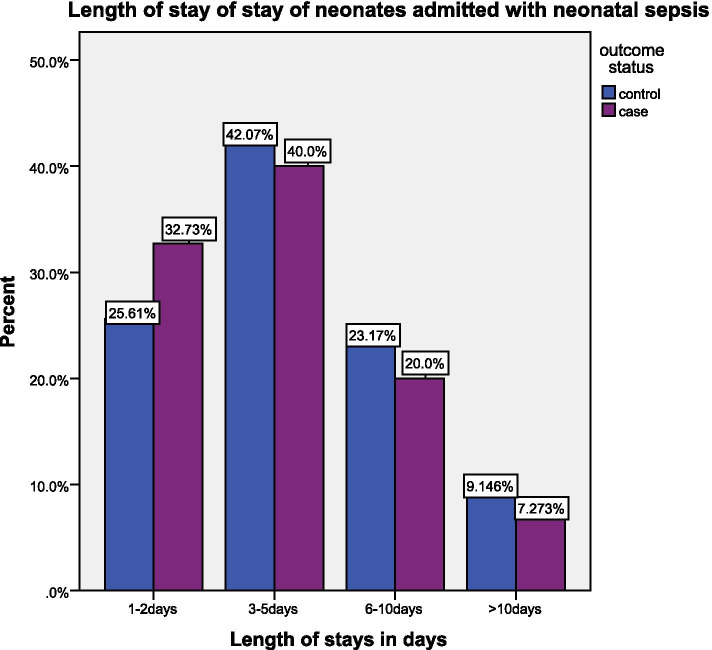
Distribution of the neonates by their length of stay at Durame General Hospital, Southern Ethiopia, 2019

In this study the proportion of neonates with first- and fifth-minute Apgar score below 7 (low Apgar score) was 27 (49.1%) and 16 (29.1%) in cases which were higher than controls 45 (27.4%) and 17 (10.4%) respectively. Among clinical features, most neonates 46 (83.6%) of cases and 106 (64.6%) of the controls had poor feeding at presentation. More than half of the cases 30 (54.5%) were presented with a hypothermic appearance of their body temperature whereas most of the controls 81 (49.4%) were presented with normal body temperature. Regarding associated comorbidities, perinatal asphyxia is more common among the cases 12 (54.5%) and controls 16 (38.1%) than the others (Table 3 and Fig. 1).

**Table 3:** Description of neonatal characteristics for the study of predictors of mortality in neonatal sepsis in Durame general hospital, Kembata tambaro zone southern Ethiopia 2019

Variable	Responses	Case Count (%)	Control Count (%)	Total
Axillary temperature	Normal body temperature	17(30.9)	81(49.4)	98(44.7)
	Hypothermia	30(54.5)	55(33.5)	85(38.8)
	Fever	8(14.5)	28(17.1)	36(16.4)
Respiratory rate	Normal RR	33(60.0)	120(73.2)	153(69.9)
	Bradypnea	13(23.6)	11(6.7)	24(11.0)
	Tachypnea	9(16.4)	33(20.1)	42(19.2)
Pulse rate	Normal PR	42(76.4)	135(82.3)	177(80.8)
	Bradycardia	8(14.5)	16(9.8)	24(11.0)
	Tachycardia	5(7.3)	13(7.9)	18(8.2)
Respiratory distress	Yes	29(52.7)	57(34.8)	86(39.3)
	No	26(47.3)	107(65.2)	133(60.7)
Poor feeding	Yes	46(83.6)	106(64.6)	152(69.4)
	No	9(16.4)	58(35.4)	67(30.6)
Abdominal distention	Yes	7(12.7)	13(7.9)	20(9.1)
	No	48(87.3)	151(92.1)	199(90.9)
Skin color	Poor	9(16.4)	19(11.6)	28(12.8)
	Good	46(83.6%	145(88.4)	191(87.2)
Have jaundice	Yes	8(14.5)	18(11.0)	26(11.9)
	No	47(85.5)	146(89.0)	193(88.1)
Have convulsion	Yes	11(20.0)	14(8.5)	25(11.4)
	No	44(80.0)	150(91.5)	194(88.6)
Fist minute APGAR score	Low score	27(49.1)	45(27.4)	72(32.9)
	Normal score	28(50.9)	119(72.6)	147(67.1)
APGAR5 score category	Low score	16(29.1)	17(10.4)	33(15.1)
	Normal score	39(70.9)	147(89.6)	186(84.9)
Comorbid disease	Yes	22(40.0)	42(25.6)	64(29.2)
	No	33(60.0)	122(74.4)	155(70.8)
Meconium aspiration syndrome	Yes	7(33.3)	16(37.2)	23(35.9)
	No	14(66.7)	27(62.8)	41(64.1)
Congenital abnormality	Yes	1(4.8)	4(9.3)	5(7.8)
	No	20(95.2)	39(90.7)	59(92.2)
Perinatal asphyxia	Yes	12(54.5)	16(38.1)	28(43.8)
	No	10(45.5)	26(61.9)	36(56.2)
Other co morbidities	Yes	8(38.1)	15(34.9)	23(35.9)
	No	13(61.9)	28(65.1)	41(64.1)
WBC count	Normal count	28(50.9)	105(64.0)	133(60.7)
	Leucopenia	7(12.7)	12(7.3)	19(8.7)7%
	Leukocytosis	20(36.4)	47(28.7)	67(30.6)
Platelet count	Normal count	35(63.6)	89(54.3)	124(56.6)
	Thrombocytopenia	12(21.8)	64(39.0)	76(34.7)
	Thrombocytosis	8(14.5)	11(6.7)	19(8.7)
Neutrophil count	Normal count	37(67.3)	123(75.0)	160(73.1)
	Neutropenia	2(3.6)	5(3.0)	7(3.2)
	Elevated count	16(29.1)	36(22.0)	52(23.7)
Blood glucose	Normal level	45(81.8)	144(87.8)	189(86.3)
	Hypoglycemia	6(10.9)	11(6.7)	17(7.8)
	Hyperglycemia	4(7.3)	9(5.5)	13(5.9)
Total		55	164	219

### Predictors of sepsis related neonatal mortality

In bivariate analysis, all variables which had a *p*-value of less than 0.25 such as the age of the neonates, parity of the mother, poor feeding, hypothermia, convulsion, body weight, estimated gestational age, APGAR score at the first and fifth minute, comorbidities, respiratory distress, thrombocytopenia, thrombocytosis, leucopenia, and leukocytosis were collectively entered in the multivariable analysis. In multivariable logistic regression analysis, poor feeding, respiratory distress, convulsion, and estimated gestational age were found to be predictors for the occurrence of death after controlling possible confounders.

The multivariable logistic regression result showed that the odds of sepsis-related mortality among neonates who had history of poor feeding were about 4 times higher than those neonates who did not have history of poor feeding [AOR = 4.15; 95% CI (1.64, 10.49). This study showed that neonates who had respiratory distress had 2.7 times higher odds of sepsis-related mortality compared to those neonates who did not have respiratory distress [AOR =**2**.72; 95% CI (1.31, 5.61), *p*-value = 0.007]. The odds of sepsis-related mortality among the neonates with history of convulsion was three times higher than those neonates who did not have history of convulsion [AOR =3.13; 95% CI (1.12, 8.76)]. This study also showed that newborn infants delivered before 37 completed weeks of gestation (preterm babies) were at risk for sepsis-related neonatal mortality. Neonates who delivered before 37 completed weeks of gestation (preterm babies) had 5 times higher odds of mortality compared to neonates who were born after 37 completed weeks of gestation [AOR = 4.64; 95%CI (2.17, 9.91)] (Table 4).

**Table 4:** Bivariate and multivariate logistic regression analyses of sepsis-related neonatal mortality among neonate admitted in NICU at Durame General Hospital, Southern Ethiopia, 2020

Variables	Responses	Case Count (%)	Control Count (%)	COR	AOR(95%CI)
Respiratory distress	Yes	29(52.7)	57(34.8)	2.09	**2**.72(1.31, 5.61)
	No	26(47.3)	107(65.2)	1	1
Estimated gestational age	< 37 weeks	33(60)	46(28)	3.85	4.64(2.17, 9.9)
	≥37 weeks	22(40)	118(72)	1	1
Convulsion	Yes	11(20.0)	14(8.5)	2.68	3.13(1.12, 8.76)
	No	44(80.0)	150(91.5)	1	1
Poor feeding	Yes	46(83.6)	106(64.6)	2.79	4.15(1.64, 10.49)
	No	9(16.4)	58(35.4)	1	1

## Discussion

This study attempted to look for determinants of sepsis-related mortality by incorporating as many risk factors as possible. The findings of multivariate logistic regression analysis of this study identified history of respiratory distress, poor feeding, estimated gestational age less than 37 weeks (prematurity), and convulsion as determinants of mortality in neonatal with sepsis.

This study observed a statistically significant association of clinical presentation like respiratory distress with the risk of sepsis-related mortality. Specifically, neonates with history of respiratory distress had approximately three times higher odds of sepsis-related mortality compared with those neonates who did not have this clinical manifestation. This finding is supported by previous studies conducted at Duhok city in Iraq and Nigeria [16–18]**.** This might be due to the fact that babies with respiratory distress don’t have a protein called surfactant that keeps small air sacs in the lungs from collapsing which increases the risk of neonatal mortality.

In this study, the majority (83.6%) of the cases had history of poor feeding at presentation with four times higher odds of sepsis-related death compared to neonates who were feeding adequately. This finding is in line with the previous finding reported in Thailand, which showed that neonatal death is about 8 times more likely in patients with clinical signs of poor feeding compared with the good ones [19]. A study conducted in India also revealed feeding as a protective factor of mortality in babies with neonatal septicemia [20]. This might be explained by the fact that in the newborn period poor feeding or inadequate caloric intake is likely to produce hypoglycemia. Endotoxaemia and sepsis have been shown to produce hypoglycemia by inhibition of gluconeogenesis, lactic acidosis, and increased glucose requirements. Besides, breast milk is a source of vitamin A and antibodies that help to fight infections. Thus, the risk of death might be elevated in this group of neonates.

Estimated gestational age less than 37 weeks (prematurity) had shown a significant association with the risk of sepsis-related neonatal mortality with the likelihood of death of 4.6 times higher among neonates born before 37 completed weeks of gestation compared to those neonates born thereafter. This result is consistent with studies conducted in Indonesia, Thailand, Duhok city in Iraq, in central India, south-eastern Mexico, and a systematic review conducted in developing countries [18, 19, 21–24]. This might be explained by the fact that premature infants are at increased risk for developing complications of septicemia because of deficiencies in humoral and cellular immunity.

This study revealed that the odds of sepsis-related mortality were about 3 higher among the neonates with a history of convulsion compared to those neonates who didn’t have this clinical sign. This result is in agreement with previous reports that associate this factor with poor prognosis and death in the neonate with infection [17, 21, 25]. Neonatal convulsion increases structural brain lesions that include hemorrhage (intracerebral, subarachnoid, and intraventricular) and infarctions of the brain which affects the overall physiological and hemodynamic stability. Another possible reason for increases in an acute outcome like mortality is an acute neonatal encephalopathy (includes classic hypoxic-ischemic encephalopathy).

This study was limited by the fact that it is a retrospective review of neonatal records and laboratory reports. As such, data collection was restricted to information previously recorded and this may be incomplete for some of the relevant variables under review. This study also lacks data on microorganisms including culture findings, drug resistance, and sensitivity pattern. Additionally, since the study was conducted on only admitted neonates in a single hospital excluding those that were referred to other hospitals, the results might lack generalizability to the total population of sepsis cases.

## Conclusion

The findings of this study noted that in septicemic neonates admitted in neonatal intensive care units (NICU), respiratory distress, and poor feeding, estimated gestational age less than 37 completed weeks (prematurity) and convulsion were significantly associated with sepsis-related neonatal mortality. Therefore, there is a need to closely monitor preterm babies for features of sepsis and to commence adequate therapeutic supports in addition to appropriate antibiotic therapies for them. Early detection and appropriate management of patients’ presentation like respiratory distress, poor feeding, and convulsion are necessary to reduce sepsis-related neonatal mortality. Blood glucose needs to be frequently monitored in infants admitted with sepsis especially in those with history of poor feeding to allow prompt management of possible hypoglycemia. Similarly, primary care organizations should increase their support towards maternal education and incorporate routine neonatal sepsis screening into the care of neonates considering the identified predictors of mortality and should equip their NICUs’ with adequate and appropriate ventilation supports to help neonates with respiratory distress.

## Data Availability

Data will be available upon request from the corresponding author.

## Abbreviations

ANC: Antenatal care
APGAR: Appearance, pulse, grimace, activity, respiration
CBC: Complete blood count
DGHD: Durame general hospital
EDHS: Ethiopia demographic and health survey
EGA: Estimated gestational age
EONS: Early onset neonatal sepsis
